# Acupuncture combined with immunotherapy for recurrent and metastatic cervical cancer: a pilot RCT protocol

**DOI:** 10.3389/fonc.2026.1718170

**Published:** 2026-01-28

**Authors:** Yan Wang, Yi Xie, Yuhang Fang, Bailu Sui, Xinhe Yuan, Yu Chen, Ying Zhang

**Affiliations:** 1 Department of Oncology, Guang’anmen Hospital, China Academy of Chinese Medical Sciences, Beijing, China; 2 Graduate School, Beijing University of Chinese Medicine, Beijing, China

**Keywords:** acupuncture, immune checkpoint inhibitors, protocol, randomized controlled trial, recurrent and metastatic cervical cancer

## Abstract

**Background:**

Cervical cancer (CC) is one of the most common malignant tumors in gynecology, posing a serious threat to women’s health. Particularly for the recurrent/metastatic cervical cancer (R/M CC), the available treatment options are limited and often yield suboptimal outcomes. Although the advent of immune checkpoint inhibitors (ICIs) has brought some improvement in the efficacy for R/M CC, low response rates to ICIs remain a significant challenge in the treatment of R/M CC. In recent years, acupuncture has demonstrated certain clinical efficacy in enhancing the immune function of patients with malignant tumors. This study aims to preliminarily explore the efficacy and safety of acupuncture combined with ICIs for R/M CC and providing data support for subsequent large-scale clinical studies.

**Methods:**

The study is designed as a multicenter, open-label, randomized, and controlled clinical trial. A total of 90 eligible participants will be randomly assigned to the experimental group or the control group in a 1:1 ratio. The experimental group will receive the standard therapeutic regimen including ICIs regimen combined with acupuncture, while the control group will receive the standard therapeutic regimen including ICIs alone. Treatment will be discontinued upon completion of four cycles, disease progression, or intolerable toxicity. Follow-up assessments will be scheduled every 12 weeks until patient death, loss to follow-up, or 2 years after randomization. The primary endpoint of this study is the Objective Response Rate (ORR). Secondary endpoints include Progression-Free Survival (PFS), Overall Survival (OS), immune function, and quality of life. Additionally, detailed records of all adverse events (AEs) will be maintained.

**Discussion:**

This study will be the first randomized controlled trial to investigate the efficacy and safety of acupuncture combined with ICIs for R/M CC, aiming to explore the synergistic enhancement of ICIs by acupuncture as an adjuvant therapeutic modality.

**Clinical trial registration:**

## Background

Cervical cancer (CC) is one of the most common malignant tumors in gynecology. According to the data from GLOBOCAN 2022, the incidence and mortality of CC rank fourth among female tumors (1). Despite significant progress in the screening and prevention of CC in recent years, which has led to a decrease in the incidence and mortality of early-stage CC, the treatment outcomes for recurrent and metastatic cervical cancer (R/M CC) remain unsatisfactory, with limited therapeutic options (2). Studies have shown that the R/M CC is as high as 70%, with a median survival of only 16.8 months (3). Therefore, it is crucial to explore new therapeutic strategies for R/M CC.

Immune checkpoint inhibitors (ICIs) have been applied in various solid tumors and have demonstrated a certain degree of antitumor activity (4). The results of the KEYNOTE-826 study, published in 2021, indicated that pembrolizumab can increase the objective response rate (ORR) by more than 15% and extend the median progression-free survival (mPFS) by over 2 months in R/M CC (5). Based on these findings, the Food and Drug Administration (FDA) approved the combination of pembrolizumab and platinum-based chemotherapy ± bevacizumab as the first-line treatment for programmed cell death ligand 1 (PD-L1)-positive R/M CC. This approval marked a new chapter in the ICIs of CC. Although ICIs has to some extent improved the survival of R/M CC, there is still a low response rate to ICIs in R/M CC due to immune resistance and tumor microenvironment suppression (6).

Clinical studies have confirmed that acupuncture can improve the immune function of cancer patients. Research has shown that acupuncture treatment can regulate the levels of cytokines such as interleukin-2 (IL-2), interleukin-10 (IL-10), tumor necrosis factor-α (TNF-α), and interferon-α (IFN-α), increase the level of natural killer cells (NK), macrophages, CD4^+^, CD8^+^ T cells, and B lymphocytes, and activate and enhance innate and adaptive immunity (7, 8). Clinical studies have shown that acupuncture at acupoints such as Zusanli (ST36), Guanyuan (CV4), Qihai (CV6), and Sanyinjiao (SP6) can increase the levels of CD4^+^, CD8^+^, and CD4^+^/CD8^+^ T cell subsets and NK cells, reduce pro-inflammatory cytokines such as interleukin-1β (IL-1β) and TNF-α, and thereby enhance immune function to delay tumor growth (9, 10). A meta-analysis has shown that acupuncture can improve the immune function of cancer patients and improve their prognosis (11).

Previous studies have confirmed the efficacy and advantages of acupuncture in regulating immune function. Therefore, whether acupuncture can improve the immune status of R/M CC Participant to increase the response rate to ICIs is a clinical issue that has not yet been resolved. This study can fill the gap in the efficacy of acupuncture combined with ICIs for R/M CC and explore the mechanisms of the synergistic effects of acupuncture on ICIs in R/M CC, providing evidence-based medical evidence for their treatment.

## Methods

### Objective

This pilot randomized controlled trial aims to preliminarily validate the efficacy and safety of acupuncture combined with ICIs for R/M CC, and to explore the synergistic mechanism of acupuncture in enhancing anti-tumor immune function and to provide data support for subsequent large-scale clinical studies.

### Study design

The study is a multicenter, open-label, randomized controlled clinical trial that compares the efficacy and safety of acupuncture combined with the standard treatment regimen that includes ICIs versus the standard treatment regimen with ICIs alone in R/M CC. Participants who meet the inclusion and exclusion criteria will be randomly assigned to the experimental group or the control group in a 1:1 ratio. All participants in both groups will receive the standard treatment regimen that includes ICIs. Participants allocated to the experimental group will receive acupuncture plus the standard treatment regimen that includes ICIs during each cycle (21-28 days). In contrast, the control group will receive only the standard treatment regimen that includes ICIs.

**Figure 1:**
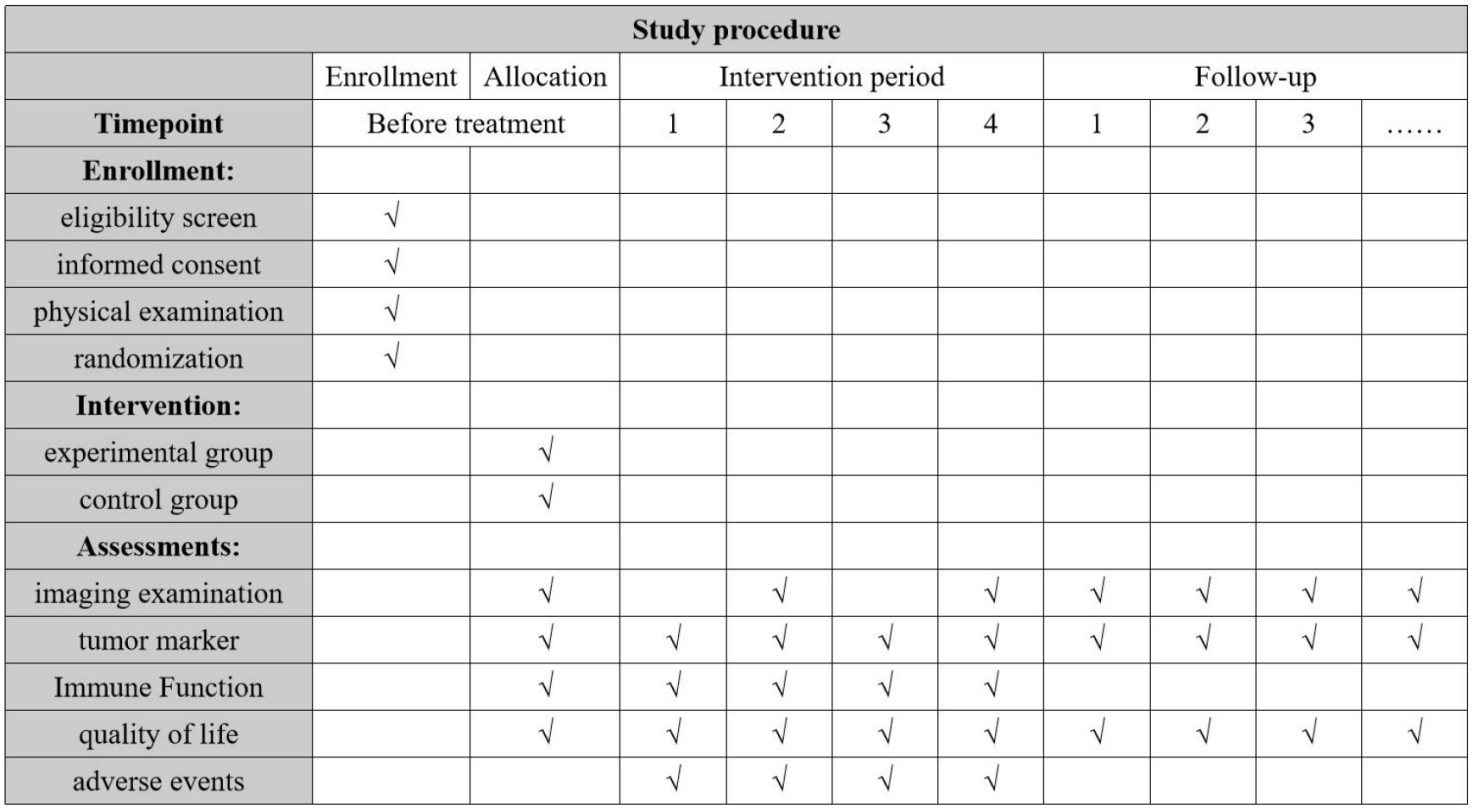
Participant timeline.

**Figure 2:**
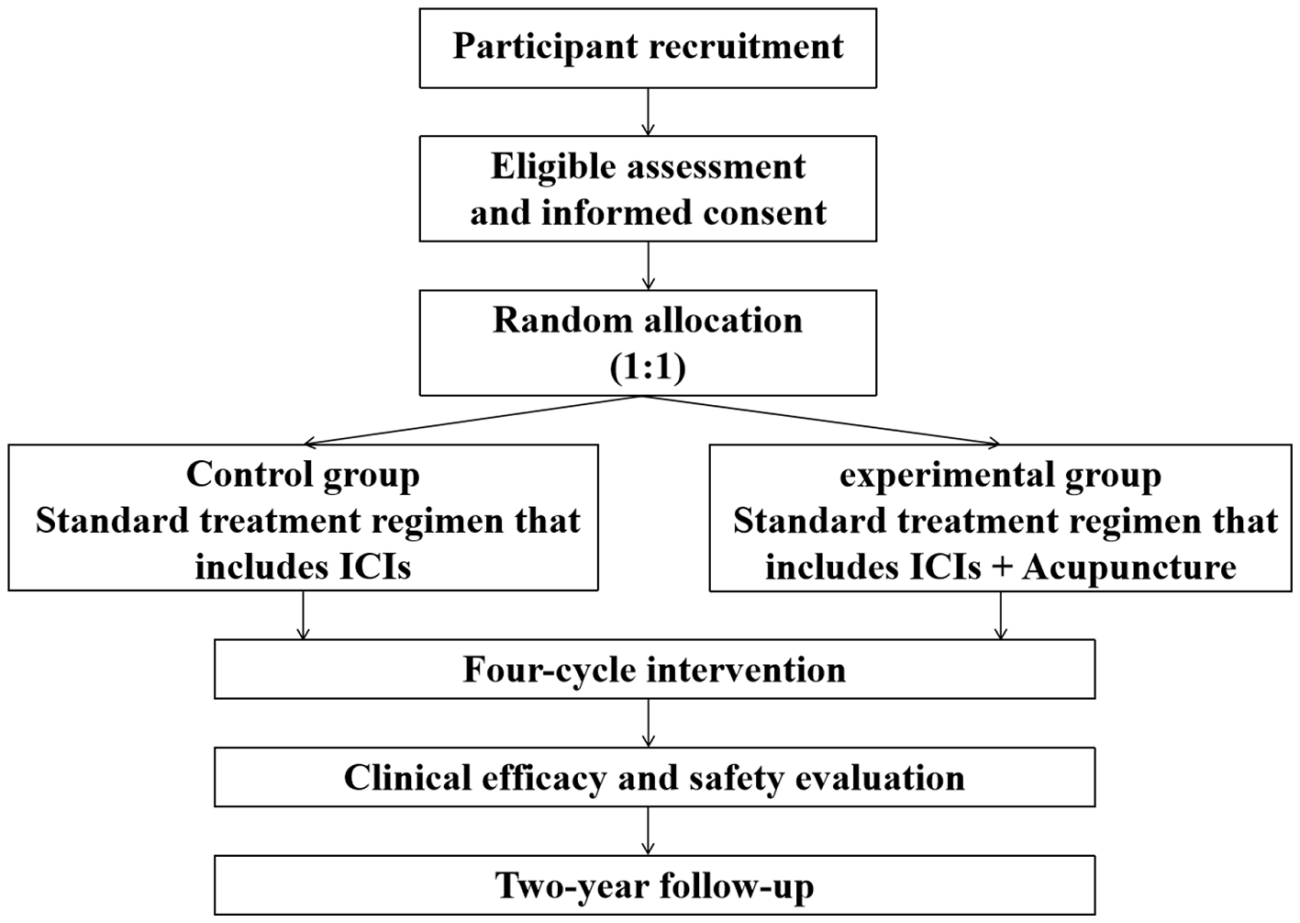
Participant enrollment process flowchart.

Treatment for eligible Participant will continue until the completion of four cycles treatment since randomization, disease progression as defined by Response Evaluation Criteria in Solid Tumors 1.1 (RECIST 1.1), the occurrence of unacceptable toxicity, or withdrawal of informed consent. Follow-up assessments will be conducted every 12 weeks until death, loss to follow-up, or 2 years after randomization. The trial has been registered on the ClinicalTrials.gov platform (ID NCT06591078). The participant timeline and trial flow diagram are shown in **Figures 1** and **2**, respectively.

### Ethical approval and patient consent

This project has been approved by the Ethics committee of China Academy of Chinese Medical Sciences Guang’anmen Hospital (2024-084-KY), Guangdong Provincial Hospital of Traditional Chinese Medicine (2024-228-01), Shanghai University of Traditional Chinese Medicine Affiliated Yueyang Hospital of Integrated Traditional Chinese and Western Medicine (2025-080), etc. Research investigators at each participating center will obtain informed consent from the candidates. Participants will be fully informed of the acupuncture procedure, possible pain, and adverse reactions, and will only participate in the study after signing the informed consent form voluntarily.

### Outcomes

#### Primary outcome

Objective Response Rate (ORR): ORR represents the percentage of Participant achieving a predefined reduction in tumor volume and maintaining this reduction for a specified minimum duration, assessed in accordance with RECIST that includes cases of complete response (CR) and partial response (PR).

#### Secondary outcomes

1. Progression-Free Survival (PFS): PFS is defined as the time from randomization to the occurrence of tumor progression or death.
2. Overall Survival (OS): OS is defined as the time from randomization until death from any cause.
3. Immune Function: Includes levels of CD3+, CD4+, CD8+, CD4+/CD8+ ratio, and NK cells.
4. Quality of Life: The European Organization for Research and Treatment of Cancer Quality of Life Questionnaire-Core 30 (QLQ-C30) and The European Organization for Research and Treatment of Cancer Quality of Life Questionnaire-Cervical Cancer Module (QLQ-CX24) will be employed to assess score changes.

#### Safety outcomes

During the period when participants receive ICIs and acupuncture treatment, a strict daily monitoring system will be implemented. Adverse events and their severity experienced by participants will be recorded (evaluated according to the National Cancer Institute Common Terminology Criteria for Adverse Events Version 5.0 (NCI CTCAE V5.0) criteria for adverse events, including but not limited to myelosuppression, hepatic and renal function impairment, skin reactions, pneumonia, colitis, nephritis, endocrine disorders, etc.

### Eligibility criteria

#### Inclusion criteria

1. Participant with squamous cell carcinoma, adenocarcinoma, adenosquamous carcinoma R/M CC that is not suitable for surgery and/or radiotherapy;
2. Age 18-70 years;
3. Have at least one measurable lesion according to RECIST 1.1;
4. Eastern Cooperative Oncology Group performance status (ECOG) performance status of 0 or 1;
5. Life expectancy exceeding 3 months;
6. Participant have normal function of vital organs, specifically:
  ① Absolute neutrophil count (ANC) ≥1.5×10^9/L;
  ② Platelet count ≥80×10^9/L;
  ③ Hemoglobin ≥90g/L;
  ④ Total bilirubin ≤1.5× upper limit of normal (ULN);
  ⑤ Aspartate aminotransferase (AST) and alanine aminotransferase (ALT) ≤2.5×ULN;
  ⑥ Creatinine ≤1.5×ULN or creatinine clearance ≥60 ml/min;
  ⑦ Baseline albumin ≥28g/L;
7. Thyroid-stimulating hormone (TSH) level ≤1×ULN;
8. Participant are able to provide written informed consent.

#### Exclusion criteria

1. Participant who are not suitable for acupuncture due to needle fainting, needle phobia, skin damage, infection, or tumor metastasis at the acupuncture site;
2. Participant who are currently participating in another clinical trial or have completed another clinical trial within the past 4 weeks;
3. Previous use of ICIs, including but not limited to other anti-programmed death-1 (PD-1) and anti-PD-L1 antibodies;
4. Known allergy to any component of the ICIs drugs;
5. Current use of immunosuppressive drugs;
6. Participant with any active autoimmune disease or history of autoimmune disease, including but not limited to hepatitis, pneumonia, uveitis, colitis;
7. Clinically significant cardiovascular disease;
8. Arterial or venous thrombosis within 6 months;
9. Poorly controlled hypertension with antihypertensive medications;
10. Not recovered from adverse events of previous medication (except alopecia);
11. Known active central nervous system metastasis;
12. Participant with a history of invasive malignancy with any evidence of disease within the past 5 years;
13. Active infection requiring systemic therapy;
14. History of immunodeficiency, including seropositivity for human immunodeficiency virus (HIV) or other acquired or congenital immunodeficiency diseases;
15. Suspected bowel obstruction or Participant at risk of vaginal rectal fistula or vaginal bladder fistula;
16. Any other medical, psychiatric, or social condition that the investigator considers may interfere with the subject’s rights, safety, welfare, or ability to provide informed consent, cooperate, and participate in the study, or may interfere with the interpretation of the results.

### Randomization, allocation concealment, and blinding

#### Randomization method

The study employs a central randomization system for allocation. Once the investigator has confirmed that a subject meets the inclusion criteria, they log into the Interactive Web Response System (IWRS) to input the subject’s unique identifier and necessary stratification information. The stratification factors are: first-line combination therapy/second-line therapy. Within each stratum, subjects are randomly assigned to the experimental and control groups using a computer-generated random sequence. The system will generate the random assignment results in real-time and inform the investigator. All assignment results are recorded in the system to ensure the transparency and traceability of the allocation process. To prevent selection bias, the assignment results are kept confidential until the subject is enrolled.

#### Allocation concealment

The study employs a central randomization system to conceal allocation. After the investigator has confirmed that a subject meets the inclusion criteria, they log into the central randomization system to input the subject’s unique identifier and necessary stratification information. The system will generate the random assignment results in real-time and inform both the investigator. All assignment results are recorded in the system to ensure the transparency and traceability of the allocation process.

#### Blinding

This is an open-label randomized trial designed to evaluate the preliminary efficacy and safety of acupuncture in ICI. Participants are randomly assigned to the experimental and control groups in a 1:1 ratio. After participants have signed the informed consent form, they are fully aware of the specific treatment they will receive. Both the participants and the researchers are aware of the specific treatment group to which the participants have been assigned.

To minimize detection and measurement bias, the radiological studies for ORR will be centrally reviewed by two committee-certified radiologists who are blinded to treatment allocation. In the event of inconsistent assessments, a third independent radiologist, who is also unaware of the treatment allocation, will be consulted for arbitration. Additionally, the quality of life assessments will be conducted and analyzed by the study coordinators and an external data management team, who are likewise unaware of the treatment allocation. These measures ensure that both objective and subjective endpoints are assessed impartially. Even though the study is of an open-label nature, these steps maximize the reduction of detection and measurement bias.

### Treatment

#### Control group

The standard treatment regimen incorporating ICIs will be administered, adhering to the systemic therapy protocol for cervical cancer outlined in the NCCN Clinical Practice Guidelines in Oncology for Cervical Cancer, Version 1.2023.

#### Experimental group

Building upon the foundation of the control group’s treatment, participants in the experimental group will additionally receive acupuncture therapy.

1. Acupuncture Needles and Apparatus: Disposable acupuncture needles (p0.3×40mm, 1.5 cun (1 cun ≈ the width of the patient’s thumb at the inter-phalangeal joint; a patient-specific proportional unit)) produced by Suzhou Medical Supplies Factory Co., Ltd. will be utilized, in conjunction with a low-frequency electronic pulse therapy device (model G6805-1A) manufactured by Shanghai Huayi Medical Instruments Co., Ltd.
2. The selection and localization of acupuncture points will be guided by the principles outlined in the national higher education traditional Chinese medicine colleges and universities planning textbook “Acupuncture and Moxibustion” (10th Edition).
3. Acupuncture Techniques:

Participant are positioned supine, and the local skin is routinely disinfected.

① Zusanli (ST36): 3 cun below the anterior superior iliac spine, lateral to the tibia, 1 cun from the anterior border of the tibia.

A 0.3×40mm filiform needle is inserted vertically to a depth of 0.5-1 cun. The needle is then manipulated with gentle, small-amplitude lifting, thrusting, and rotating movements three times until the patient feels a sensation of soreness, numbness, or distension, indicating the “deqi”. After “deqi”, the needle handles of both sides of the ST36 are connected to the electrodes of the electroacupuncture device in a transverse manner. A continuous wave is selected, with a frequency adjusted to 2 Hz and a current intensity set at 0.5-2 mA.

② Sanyinjiao (SP6): 3 cun above the medial malleolus, on the medial side of the tibia.

The needle is inserted slowly to a depth of 0.5-1 cun, avoiding nerves. If there is no electric-like sensation after insertion, the needle is manipulated with gentle, small-amplitude lifting, thrusting, and rotating movements three times until the patient feels a sensation of soreness, numbness, or heaviness, indicating the “deqi”. If an electric-like sensation occurs after insertion, the needle is lifted to the subcutaneous layer, the direction of the needle tip is adjusted, and the needle is reinserted. After reinsertion, the needle is manipulated with gentle, small-amplitude lifting, thrusting, and rotating movements three times. If the patient has urinary symptoms such as frequent urination, nocturia, urinary incontinence, difficulty in urination, lower abdominal discomfort, or perineal pain, after “deqi”, the needle handles of both sides of the Sanyinjiao points are connected to the electrodes of the electroacupuncture device in a transverse manner. A continuous wave is selected, with a frequency adjusted to 2 Hz and a current intensity set at 0.5-2 mA.

③ Guanyuan (CV4): 3 cun below the umbilicus, on the midline of the abdomen.

Qihai (CV6): 1.5 cun below the umbilicus, on the midline of the abdomen.

The needles are inserted vertically to a depth of 0.5-1 cun and manipulated with gentle, small-amplitude lifting, thrusting, and rotating movements three times until the patient feels a sensation of soreness, numbness, or distension, indicating the “deqi”.

4. Acupuncture schedule and frequency:

① Frequency: One to two acupuncture sessions per week.
② Needle retention: Each session involves needle retention for 30 minutes.
③ Minimum treatments per cycle: At least four acupuncture treatments must be completed per cycle.
④ Cycle duration: Each cycle lasts for 21 days.
⑤ Total treatment duration: The entire treatment course consists of four cycles.

The acupuncture schedule is determined based on previous clinical studies and pharmacokinetic characteristics of immune checkpoint inhibitors (ICIs) (12, 13). A randomized controlled trial (RCT) showed that participants who received acupuncture/electro-acupuncture at a frequency of ≥1 session per week before or concurrently with ICI therapy experienced a median PFS extension from 7.87 to 10.23 months (12). In addition, the 21-day cycle is consistent with the administration cycle of ICIs (e.g., pembrolizumab is administered every 21 days), ensuring the synergistic effect of acupuncture and immunotherapy.

5. Prohibition on medication and treatment:

The use of any antitumor therapy other than standard treatment is prohibited, including non-palliative radiotherapy; traditional Chinese medicine (TCM) injections, TCM decoctions and TCM patent medicines that with antitumor effects; other treatment modalities not specified in the protocol.

During the acupuncture process, a professional TCM physician with more than 10 years of clinical experience will operate, strictly controlling the depth (avoiding deep insertion into blood vessels and organs) and manipulation (gentle twisting, lifting, and thrusting with amplitude on0° to reduce pain). The needle insertion depth must within the safe range recommended by the WHO Acupuncture Guidelines (14) and for the acupuncture manipulation “deqi” (a mild sour, numb, and distending sensation, but not “immense pain”) is considered appropriate.

Meanwhile, use the Numerical Rating Scale (NRS) to evaluate patients’ pain degree before, during, and after each acupuncture session. If the pain score ≥4 points, immediately adjust the manipulation or stop the operation.

### Sample size

Given that the clinical efficacy of ICIs in CC warrants further enhancement and that prior research has indicated acupuncture’s potential to bolster the immune function of cancer participant, this exploratory randomized controlled trial is not designed to test efficacy. A total of 90 participants (45 per group) were selected to provide sufficient precision for estimating the median ORR and variance while maintaining logical feasibility. This aligns with published recommendations (15-25 per group) (15–17).

Assuming an anticipated ORR of 30-35% in the experimental arm, 45 participants per group yield a 95% Wilson score interval of ± 13-14%, which meets the precision criteria for phase II exploratory oncology trials (18). This sample size aligns with methodological recommendations of 30-50 participants per arm for estimating response rate variance in pilot studies (18). Considering our center’s enrollment capacity of 30-40 cervical cancer patients receiving ICIs annually and a conservative 10% attrition rate derived from prior ICI trials (19), 45 participants per arm ensure ≥40 evaluable cases. While this exploratory design (n=90) is not powered for definitive efficacy testing, it provides robust preliminary data on ORR patterns and feasibility to inform subsequent confirmatory trials.

The sample size per arm was calculated using the Wilson-score‐based precision formula for a single proportion, assuming an expected ORR of 35% and requiring a half-width ≤ 13% at 95% confidence:

n = 4 × ORR(1 – ORR)/w², which yields 45 participants per group after allowing for 10% attrition.

### Data collection and management

#### Data collection

The research team will be responsible for the collection and management of data. Standardized Case Report Forms (CRFs) will be used to record all data related to the study. These CRFs will be designed in accordance with the study protocol to ensure that all necessary data points are captured. An Electronic Data Capture (EDC) system will also be employed for data entry and management. Data from all CRFs will be regularly entered into the EDC system, with double data entry conducted to ensure accuracy.

#### Data management

To ensure the authenticity and accuracy of the study data, a designated individual at each research center will assist physicians in documenting and managing all visits and examinations and will review the CRFs to verify the information. Each center will be equipped with a dedicated file cabinet, and a specific person will be responsible for the custody of the trial documents. After the follow-up is completed, all original data will be digitized, and the initial CRFs forms will be reviewed and archived by the research team.

### Statistical methods outcomes

The SAS 9.4 statistical software was used. Efficacy endpoints were analyzed using both the full analysis set (FAS) and the per-protocol set (PPS), while adverse reactions were analyzed using the safety set (SS).

All statistical tests were two-sided, and a P value of ≤0.05 was considered to indicate a statistically significant difference. The dropout rate should not exceed 20%. Continuous data were described in terms of mean, standard deviation, median, minimum, and maximum values, while categorical data were described in terms of frequency and percentage. Continuous data were analyzed using t-tests and rank-sum tests, while categorical data were analyzed using chi-square tests and Ridit analysis.

To ensure the scientific rigor and validity of the statistical analyses, all continuous variables in this study were pre-tested for normality. Specifically, the Shapiro-Wilk test was employed for samples with a size of n ≤ 50, while the Kolmogorov-Smirnov test was used for samples with n > 50. Additionally, visual inspection of quantile-quantile (Q-Q) plots was conducted to verify the distribution characteristics of the data. Variables that conformed to a normal distribution were further tested for homogeneity of variance. Those with homogeneous variance were described as mean ± standard deviation (Mean ± SD), and between-group comparisons were performed using the independent samples t-test; variables with heterogeneous variance were analyzed using the Welch’s t-test (corrected t-test). Variables that did not follow a normal distribution were presented as median (interquartile range) [M (IQR)], and between-group comparisons were carried out using the Wilcoxon rank-sum test.

Survival data were analyzed using the Kaplan-Meier method, the Wilcoxon rank-sum test, and the log-rank test. Cox proportional hazards regression model was used for multivariate survival analysis. The Cox proportional hazards model was used to calculate hazard ratios (HR) with corresponding 95% confidence intervals (CI), adjusting for the pre-specified prognostic factors, including the number of previous treatment lines.

To ensure the authenticity and reliability of the data analysis results, the data statistics and analysis were completed by an independent statistician.

## Discussion

In the treatment of R/M CC, the application of ICIs is continuously expanding. In addition to the well-known pembrolizumab, an increasing number of ICIs have been approved for R/M CC, offering patients more options (6). These include anti-PD-1 monoclonal antibodies, such as nivolumab (20), camrelizumab (21), cemiplimab (22), and balstilimab (23), anti-PD-L1 monoclonal antibodies such as atezolizumab (24), and durvalumab (25), and combination antibodies with both anti-PD-1 and anti-CTLA-4 functions, such as cadonilimab (26), all of which have shown promise in clinical practice.

Among R/M CC, ICIs used as monotherapy or in combination with other treatment modalities such as chemotherapy or targeted therapy have demonstrated remarkable clinical efficacy and significantly improved clinical benefits for patients (27). Particularly when combined with chemotherapy, antiangiogenic drugs, or used concurrently during radiotherapy, they can exert a synergistic effect (28–30).

However, despite the progress made by ICIs in improving efficacy, the application of ICIs in the treatment of R/M CC still faces several challenges. At present, the ORR of ICIs treatment generally has an upper limit of around 30%, and the increased toxicities and side effects of combination therapies to some extent restrict the further improvement of efficacy (31). For the management of patients with recurrent and metastatic cervical cancer, how to break through this bottleneck remains an urgent problem to be solved.

Acupuncture is widely recognized as a complementary therapy for treating various malignancies through the regulation of immune responses. A study indicated that in a mouse model of breast cancer, acupuncture could enhance the efficacy of anti-PD-1 therapy by promoting CD5^+^ dendritic cells and T cell-mediated antitumor immunity (32). Acupuncture can promote the activity and number of NK cells through pathways such as β-endorphin (β-EP) (33), the hypothalamic-pituitary-adrenal axis (HPA axis) (34), and the vagus nerve (9), playing a significant role in the regulation of innate immunity. Moreover, acupuncture can also exert antitumor effects by enhancing adaptive immune functions, including upregulating immune cells with positive functions, such as Th1 and CD8^+^ T cells (35, 36), and downregulating immune cells with negative functions, such as Treg and Th2 cells (33). In addition, acupuncture has shown favorable clinical efficacy in improving cancer-related pain (37), cancer-related fatigue (38), cancer-related insomnia (39), and cancer-related emotional disorders (40) et al.

The acupuncture procedure in this study is based on the theory of traditional Chinese medicine (TCM) and modern evidence-based research. Four specific acupoints (Zusanli (ST36), Sanyinjiao (SP6), Guanyuan (CV4), and Qihai (CV6)) were be selected and that have been proven to regulate immune function and inhibit tumor progression in previous studies. As core acupoints for regulating qi-blood and tonifying primordial qi, this combination follows TCM principles of “treating both root and branch causes” and “rectify and strengthen the fundamentals”. Zusanli and Sanyinjiao synergistically enhance spleen-stomach function to boost qi-blood production, while Guanyuan and Qihai warm and tonify congenital vitality. This strategic acupoint combination is a classic prescription in traditional Chinese medicine for replenishing qi and consolidating the body’s resistance. In addition, in clinical studies investigating acupuncture’s effects on improving immune function in cancer patients, the most commonly used acupoints are Zusanli (ST36), Sanyinjiao (SP6), Guanyuan (CV4), and Qihai (CV6) (11). Studies on the regulatory mechanism of acupuncture acupoints have confirmed that needle insertion can stimulate the sensory nerve endings in the acupoints, which transmit signals to the spinal cord and brain, and regulate the immune system through the neuro-immune network (41). Zusanli (ST36) and Sanyinjiao (SP6) can activate the vagus nerve, release acetylcholine, and inhibit the production of pro-inflammatory cytokines and upregulate the expression of CD4^+^ T cells, CD8^+^ T cells, and natural killer (NK) cells by activating the cholinergic anti-inflammatory pathway (42). Guanyuan (CV4) and Qihai (CV6) can enhance the proliferation and differentiation of immune cells by activate the sympathetic nervous system (43). These acupoints are selected to specifically target the anti-tumor immune response without non-specifically activating the immune system. Clinical trials have also demonstrated that acupuncture produces measurable immunostimulation in patients with advanced cancers.

In addition, acupuncture at different regions of the body has different effects on the immune system, which is related to the innervation and meridian distribution of the acupoints. Limb acupoints, such as Zusanli (ST36) and Sanyinjiao (SP6), mainly regulate the systemic immune function by activating the cholinergic anti-inflammatory pathway and abdominal acupoints such as Guanyuan (CV4) and Qihai (CV6), mainly regulate the local immune function of the abdomen by activating the sympathetic nervous system (44). Therefore, in this study, four acupoints from different regions (limbs, abdomen) were be selected to achieve a comprehensive regulation of the immune system.

In summary, acupuncture regulates immune function through a variety of mechanisms. It not only enhances the antitumor efficacy but also alleviates patients’ symptoms and improves their quality of life. The synergistic effect of acupuncture with modern medical treatments offers new ideas and methods for the treatment of malignancies. Therefore, acupuncture holds significant clinical value in the treatment of immune-related diseases, including malignancies.

To ensure the reliability of the study results, we will implement quality control measures from four different aspects: 1) Each center must designate a person to be in charge of the overall coordination of the process. 2) Training will be provided to the researchers before the project begins. 3) Double validation of CRFs and EDC system to ensure the accuracy and completeness of the data. 4) Regular monitoring will be conducted.

To the best of our knowledge, this is the first prospective, randomized, controlled clinical study of acupuncture combined with ICIs for the R/M CC. We hope that this trial will initially provide some clinical evidence for the efficacy and safety of acupuncture combined with ICIs in the treatment of R/M CC, provide data support for subsequent large-sample clinical studies, and gradually extend to multiple types of cancer.

Concomitantly, in future subsequent large-sample clinical studies, immune indicators of the tumor microenvironment (TME) can be incorporated to further elucidate the potential mechanisms underlying acupuncture’s therapeutic effects, such as PD-L1, key cytokines, immunometabolic molecules, and cells of the innate and adaptive immune system. Accumulating evidence has confirmed that the TME plays a pivotal role in tumor progression, metastasis, and immune escape (45, 46), while acupuncture has been consistently demonstrated to regulate immune cell function and modulate inflammatory responses across multiple disease models (7, 47). Such investigations will not only deepen the mechanistic understanding of acupuncture in tumor therapy but also provide potential targets for optimizing personalized acupuncture regimens for cancer patients.

## Limitations

This study design has several limitations. Firstly, this study is designed as a small-scale exploratory trial with a primary focus on laying a foundational evidence base and generating critical effect-size data to inform the design and sample size calculation of subsequent large-scale RCTs. Given the novelty of the intervention under investigation and the lack of prior empirical data on its efficacy and safety in the target population, this preliminary study aims to address key knowledge gaps, including the feasibility of study procedures, participant recruitment and retention rates, and preliminary estimates of the intervention’s potential therapeutic effects. By evaluating these critical parameters, the study seeks to mitigate risks associated with large-scale RCTs, such as impractical study designs, inadequate statistical power, or unforeseen implementation challenges.

Secondly, a key limitation of this study lies in the narrow population representativeness of the participants, as all enrolled subjects were of Chinese ethnicity. This may, to a certain extent, compromise the external validity of the study findings. The potential impacts of this limitation are primarily reflected in two aspects. On the one hand, compared to other ethnic origins, Chinese populations may exhibit differences in genetic polymorphisms associated with immune regulation and disease susceptibility, such as the cytotoxic T-lymphocyte associated antigen-4 (CTLA-4) gene variant rs5742909 (48). These genetic characteristics are likely to modulate the physiological response mechanisms and therapeutic outcomes of acupuncture interventions. On the other hand, from a sociocultural and medical perception standpoint, as an integral component of traditional Chinese medicine, acupuncture enjoys higher acceptance and awareness among the domestic population. Participants may develop certain psychological expectancy effects (e.g., placebo effects) due to cultural identification. Future studies should incorporate genetic stratification to elucidate genotype-acupuncture associations for personalized treatment.

Thirdly, a notable limitation of this study is open-label design, which stems from the inherent challenges of implementing effective blinding in acupuncture trials.

We pre-specify multi-level controls: 1) Procedural: blinded independent central imaging review, blinded biomarker identifications, and an independent Endpoint Committee; 2) Analytical: sensitivity analysis comparing investigator, adjusting for baseline optimism and concomitant medication indices, and per-protocol analysis excluding <80% acupuncture adherence; 3) Interpretation: only ORR differences >15% (95%CI lower bound >8%) will qualify as true signals. This design is justified for a signal-seeking exploratory trial emphasizing feasibility. Despite these efforts, the open-label nature of the study may still have introduced residual biases. Future studies may consider adopting more sophisticated sham acupuncture protocols (e.g., using non-penetrating needles, controlled depth of insertion) or combining objective measurement techniques (e.g., functional imaging, electrophysiological monitoring) to enhance blinding feasibility and further reduce the impact of placebo and expectation biases.

## Data Availability

The original contributions presented in the study are included in the article/supplementary material. Further inquiries can be directed to the corresponding author.
